# Seroprevalence of hepatitis B virus co-infection among HIV-1-positive patients in North-Central Nigeria: The urgent need for surveillance

**DOI:** 10.4102/ajlm.v8i1.622

**Published:** 2019-06-27

**Authors:** Terver M. Akindigh, Abba O. Joseph, Christiana O. Robert, Ocheme J. Okojokwu, Juliet N. Okechalu, Joseph A. Anejo-Okopi

**Affiliations:** 1 Infectious Diseases Unit, Jos University Teaching Hospital, Jos, Nigeria; 2 Department of Microbiology, Faculty of Natural Sciences, University of Jos, Jos, Nigeria

**Keywords:** Antiretroviral therapy, Alanine aminotransferase, CD4+ T-lymphocytes, hepatitis B virus, HIV, Serological markers

## Abstract

We report the seroprevalence of hepatitis B surface antigen among HIV-positive patients at a clinic in North-Central Nigeria. Screening for hepatitis B virus was based on serological markers. Alanine aminotransferase levels and CD4+ T-lymphocyte counts were compared between patients. The study showed that 9.2% were positive for hepatitis B surface antigen with significant differences between alanine aminotransferase levels of patients with and without hepatitis B virus. We recommend a national surveillance system to monitor control efforts.

## Introduction

Hepatitis B virus (HBV), a circular DNA virus with humans serving as the only reservoir^1^, remains a worldwide public health problem. Transmission of the virus occurs via exposure to infected blood and body fluids.^1^ In areas where the prevalence of hepatitis B surface antigen (HBsAg) is more than 8%, there are two broad possibilities for how transmission can occur: either it happens vertically at birth from chronically infected mothers to their newborns or horizontally during childhood or in adulthood.^2,3^ The infection caused by the virus is among the leading causes of liver disease, with liver-related deaths occurring in an estimated 25% – 30% of the population of chronic carriers, principally cirrhosis and hepatocellular carcinoma.^4^

A fifth of the current global population is estimated to be seropositive for HBsAg.^5^ As at 2013, an estimated 13.6% of the Nigerian population had been reported to be chronic carriers of HBsAg.^6^ Consequently, the burden of disease is considered to be high, as evidenced by the high incidence of morbidity and mortality associated with the virus.^7^

The similarity in transmission routes for HBV and HIV makes co-infection very common.^8^ HBV/HIV co-infection has been shown by some studies to increase morbidity and mortality when compared to HIV mono-infection.^9,10^ It has been reported that co-infection facilitates replication and decreases clearance of both viruses, as a result of impaired innate and adaptive immune responses.^11,12^

The HIV-positive sub-population deserves attention, considering the enormous public health consequences that co-infection with HBV can have. This cross-sectional study reports the prevalence of HBV infection among HIV-positive patients receiving treatment and care from the Jos University Teaching Hospital (JUTH), Infectious Diseases Unit.

## Methods

### Ethical considerations

Approval of the study protocol was obtained from the Institutional Ethics Committee at JUTH (study approval number, JUTH/DCS/ADM/127/XIX/5967).

### Study population

This laboratory-based study was carried out by examining 120 HIV patients older than 18 years on highly active antiretroviral therapy accessing care at JUTH who provided written informed consent. The sample size was determined using the following formula:^13^

$$\text{N}={\text{z}}^{2}\text{pq}/{\text{e}}^{2}$$ [Eqn 1]

where

z = Alpha at 95% confidence level = 1.96

p = prevalence of hepatitis B virus infection^14^ = 7.2% = 0.072

q = 0.928

e = margin of error = 0.05

N = minimum sample size = 10.2 with 10% attrition = 10.2, thus *N* = 20

### Collection of patients’ blood

Whole blood samples were obtained from each patient and used for screening for HBV serological markers as well as for performing cluster of differentiation CD4+ T-lymphocyte enumeration and clinical chemistry analysis of alanine aminotransferase (ALT). The process involved collection of blood using a BD Precision Glide™ into a 4 mL EDTA BD Vacutainer^®^ (Becton Dickinson, Plymouth, United Kingdom) for CD4+ T-lymphocyte enumeration and into a 6 mL BD Vacutainer^®^ Clot Activator Tube (Becton Dickinson, Plymouth, United Kingdom).

### Serological marker assay

Plasma was obtained by centrifuging collected whole blood at 2500 rpm for 1 min and testing for hepatitis B serologic markers, including the surface antigen (HBsAg), surface antibody (Anti HBs), envelope antigen (HBeAg), envelope antibody (Anti HBe) and core antibody (Anti HBc) of viral particles as performed using the Rapid HBV COMBO test kit (Acumen diagnostics Incorporated, China) according to the manufacturer’s instructions.

### CD4+ T-lymphocyte enumeration

CD4+ T-lymphocyte counts for each patient were obtained by adding 20 *µ*L of the patient’s whole blood to 20 *µ*L of CD4+ easy count kit monoclonal antibody, then incubating in the dark for 15 min. Afterwards, 800 *µ*L of CD4+ easy count kit no lyse buffer was added to the incubated mixture and analysed using a Partec CyFlow cytometer (Sysmex-Partec, Munster, Germany).

### Evaluation of alanine aminotransferase

ALT levels were obtained using a COBAS C311 (Roche Diagnostics, Mannheim, Germany) chemistry auto-analyser. The method involved using an Eppendorf 5708 centrifuge to spin the blood sample and obtain an aliquot of serum, which was then placed in a sample cup and processed using the COBAS C311 auto-analyser to determine the serum ALT levels of patients.

### Data analysis

Data were analysed using SPSS version 17 (IBM Corporation, Armonk, New York, United States) to obtain descriptive statistics, evaluate associations between socio-demographic variables and positivity for HBV. We also determined whether there was a difference between ALT and CD4+ counts using an unpaired *t*-test for the mean values of both groups. Decisions on statistical significance were made at a 5% level of significance.

## Results

Out of the 120 patients on highly active antiretroviral treatment who participated in the study, 88 (73.3%) were women and 32 (26.7 %) were men (Table 1). Participants in this study had a mean age of 40.4 ± 10.6 years with male participants significantly older than their female counterparts in the study. Similarly, the ALT levels of male participants were significantly higher than female participants.

**TABLE 1:** Patient characteristics by sex, Jos University Teaching Hospital HIV clinic, August 2015.

General characteristics	Men	Women	*p*
**Sex**			
*N*	32	88	-
%	26.7	73.3	-
**Age in years**			
Mean ± SD	47.2 ± 11.5	37.9 ± 9.1	< 0.0001
**CD4+ cell count (cells/µL)†**			
Mean ± SD	188.1 ± 168.1	237.8 ± 191.4	0.199
**ALT level (IU/mL)†**			
Mean ± SD	35.6 ± 28.2	23.7 ± 21.9	0.02

† , unpaired *t*-test.

ALT, Alanine aminotransferase; SD, Standard deviation.

Among the HIV-positive patients in the study, 11 were positive for HBsAg (9.2%) (Table 2). For the other serological markers in the study, 15 patients (12.5%) were positive for Anti HBs 1 patient (0.8%) was positive for HBeAg, 27 patients (22.5%) were positive for Anti HBe, and 32 patients (26.7%) were positive for Anti HBc.

**TABLE 2:** Seroprevalence of hepatitis B serological markers? among HIV-positive adult patients attending the Jos University Teaching Hospital HIV clinic, August 2015.

Serological marker	Positive		Negative	
	*n*	%	*n*	%
Hepatitis B surface antigen	11	9.2	109	90.8
Hepatitis B surface antibody	15	12.5	105	87.5
Hepatitis B envelope antigen	1	0.8	119	99.2
Hepatitis B envelope antibody	27	22.5	93	77.5
Hepatitis B core antibody	32	26.7	88	73.3

Being in the 40–49-year age group was significantly associated with HBV/HIV co-infection (*p* = 0.019) (Table 3). Our study reported a higher rate of HBV infection among the male HIV patients (12.0% versus 7.9% in female patients), but this observation was not significant (*p* = 0.494). The associations of HBV infection with age, educational level and marital status were also not statistically significant. No association between infection and marital status *(p* = 0.875) when they were compared to patients who were negative for HBsAg.

**TABLE 3:** Socio-demographic characteristics and laboratory parameters of HBV infection among HIV-positive adult patients, Jos University Teaching Hospital HIV clinic, August 2015.

Variable	Positive	Negative	*p*
**Sex**‡			
Men			
*n*	4	28	
%	12.5	87.5	0.494
Women			
*n*	7	81	
%	8.0	92.0	
**Age in years**†			
Mean ± SD	39.8 ± 6.3	40.9 ± 10.9	0.627
Median	42	40	
**Age group (years)**‡			
≤ 29			
*n*	1	18	
%	5.3	94.7	
30–39			
*n*	2	34	
%	5.6	94.4	0.019
40–49			
*n*	8	31	
%	20.5	79.5	
≥ 50			
*n*	0	26	
%	0.0	100.0	
**Educational level**‡			
No education			
*n*	1	9	
%	10.0	90.0	
Primary education			
*n*	5	28	
%	15.2	84.8	0.242
Secondary education			
*n*	4	35	
%	10.3	89.7	
Tertiary education			
*n*	6	32	
%	15.8	84.2	
**Marital status**‡			
Married			
*n*	5	59	
%	7.8	92.2	
Single			0.875
*n*	2	12	
%	14.3	85.7	
Widowed			
*n*	3	24	
%	11.1	88.9	
**CD4+ cell count (cells/*µ*L)†**			
Mean ± SD	215.7 ± 135.4	220.3 ± 188.7	0.938
Median	214	157	
**ALT level (IU/mL)**†			
Mean ± SD	42.0 ± 37.5	25.3 ± 22.4	0.033
Median	29.9	18.4	

Note: Fifteen participants did not provide a response for marital status.

ALT, Alanine aminotransferase; CD4+, cluster of differentiation.

† , unpaired *t*-test.

‡ , Chi-square distribution.

The mean CD4+ counts of HIV/HBV co-infected patients were lower than patients who were only positive for HIV; this difference was not statistically significant (*p* = 0.938) (Table 3). However, the mean ALT values for patients positive for HBsAg were significantly higher than for patients negative for HBsAg (*p* = 0.033).

## Discussion

This present study observed a 9.2% sero-prevalence of HBsAg among HIV-positive patients attending the HIV treatment clinic at JUTH. This is slightly lower than the 12.5% prevalence reported from north-western Nigeria in 2013^15^ and the 11.5% observed in 2012 another health facility in North-Central Nigeria.^16^ However, another study conducted in southwestern Nigeria reported a much lower prevalence of 5.7% among HIV-positive patients in 2009.^17^ These reports which show differences in regional prevalence may point to different epidemiological factors at work in different parts of the country and reveal a gap in our current understanding of the epidemiology of HIV/HBV co-infection. National surveillance systems to provide a full picture of the co-infection landscape are urgently needed to identify epidemiological trends over time to make sure suitable prevention measures are deployed.

Our results agree with the conclusions of a recent meta-analysis by Musa and colleagues^6^ that showed that in Nigeria, there is a continued decline in prevalence of hepatitis B infection; they estimated that the rate of decline has been approximately 0.8% annually. While our finding provides significant feedback regarding the impact of the HBV vaccination programme in Nigeria, the result remains higher than the 8% threshold that the World Health Organization considers endemic among HIV-positive individuals. We made an observation similar to other studies regarding men having higher rates of infection than women.^17,18^ The report of a recent study by Akhtar et al.^19^ in a tertiary hospital in Malaysia and another report from north-east Nigeria^20^ for HBV co-infection in HIV patients agree with our observation of an age-specific association in patients aged 40–49 years. A global age-specific prevalence, which is highest in sub-Saharan Africa, has been reported from a systematic review of data on worldwide prevalence collected over a 27-year period.^21^ The association between increasing age and being positive for HBsAg by several workers reinforces the current paradigm that the epidemiology of HBV in Nigeria is primarily via horizontal transmission.^22^ In the present study, serum ALT levels in patients who were positive for HBsAg were significantly higher than ALT levels in patients who were HBsAg-negative, which is a comparable observation to that made by Otegbayo et al.^23^ It is possible that the patients in our study population were in the late reactivation phase where high serum ALT levels accompany progressive liver damage.^24^ There is a growing interest in understanding serum HBsAg levels across the different phases of HBV infection.^25^ However, our inability to quantify HBsAg titers and ascertain the different phases of infection are limitations of our study. Although two studies documenting the natural history of the infection in Asian and European cohorts reported no significant association between ALT activity and serum HBsAg titers,^26,27^ an African perspective for the different phases may reveal a different picture in the course of infection.

### Conclusion

Despite the overall decline in HBV prevalence, it remains endemic in HIV-positive populations and requires effective surveillance to fully understand the epidemiology of HIV/HBV co-infection in Nigeria. Increased uptake of a vaccination programme among HIV-positive patients who are susceptible to HBV infection, along with the identification of patients with HBV infection for treatment, are critical strategies in prevention and control activities.
